# Experiences of physiotherapists considering virtual reality for shoulder rehabilitation: A focus group study

**DOI:** 10.1177/20552076241234738

**Published:** 2024-02-26

**Authors:** Beate Dejaco, Niamh Brady, Anne Tankink, Jeremy Lewis, Harry van Goor, J Bart Staal, Niki Stolwijk

**Affiliations:** 1 Radboud Department of Surgery, Radboud University Medical Center, Nijmegen, The Netherlands; 2 Research Group Musculoskeletal Rehabilitation, HAN University of Applied Sciences, Nijmegen, The Netherlands; 3 Department of Physiotherapy, Sports Medical Centre Papendal, Arnhem, The Netherlands; 4 Discipline of Physiotherapy, University College Cork, Cork, Ireland; 5 Evolve Health, Cork, Ireland; 6 Therapy Department, Central London Community Healthcare National Health Service Trust, London, UK; 7 Musculoskeletal Research, Clinical Therapies, University of Limerick, Limerick, Ireland

**Keywords:** Virtual reality, shoulder rehabilitation, physiotherapy, qualitative interpretive design

## Abstract

**Introduction:**

Shoulder pain is common and associated with substantial morbidity. Different treatment strategies are being prescribed with equivocal results. Virtual reality (VR) is a novel technology and emerging research suggests that VR may be a promising alternative to current treatments. Prior to effectiveness research or any large-scale introduction, VR-applications require appropriate scrutiny including feasibility- and acceptability of clinicians and patients. Therefore, the aim of this study was to collect experiences of physiotherapists after using immersive VR.

**Methods:**

A qualitative interpretive design was used to explore physiotherapists’ experiences related to the use of VR for people with shoulder symptoms. 17 physiotherapists were asked to use VR at home for five days prior to a focus group interview. Data from the focus group interviews were analyzed using a six-phase process of thematic analysis.

**Results:**

Three main themes were identified, each divided into subthemes. The main themes were: 1. VR as an extension of contemporary physiotherapy care: physiotherapists were positive about the potential of VR and its applicability in daily care. 2. Physiotherapist uncertainties of future care using VR: participants expressed concerns about their professional identity, particularly as patients engage in independent home exercises. 3. Physiotherapist's requirements for implementation of VR: participants shared their needs for evidence regarding the effectiveness and parameters such as frequency, dosage and intensity of the VR intervention.

**Conclusion:**

Physiotherapists were positive about VR as an intervention tool. However, they felt more knowledge is needed about parameters of VR. The findings of this study inform researchers and technology developers about optimal design of interventions and applications using VR.

## Introduction

Musculoskeletal shoulder pain is a common condition with a reported lifetime prevalence ranging between 6.7 to 66.7%.^1^ Shoulder pain is often cited as being a major contributor to interrupted sleep and as such may contribute to a cascade of morbidity.^2,3^ Exercise therapy is considered to be an essential component of the management for shoulder pain.^4–7^ Unfortunately, poor adherence to exercise therapy is an acknowledged barrier to symptom reduction.^8,9^ Recent technical progress has resulted in the development and implementation of extended reality (XR), encompassing virtual reality (VR), augmented reality (AR), and mixed reality (MR) for exercise therapy.

Initially used in the gaming industry, non-immersive, semi-immersive, and immersive VR technologies have been rapidly introduced in different areas of healthcare, including the management of musculoskeletal related pain.^10–16^ The mechanisms of how VR decreases pain and enhances function are not fully elucidated. Proposed theories include distraction,^17,18^ embodiment,^19^ manipulation of sensory input,^20,21^ graded exposure^16,22^ and reduction in anxiety.^23^ Dubé et al. have suggested that education and exercise should be included in the treatment of musculoskeletal shoulder conditions.^24^ VR can offer new possibilities in patient education, and helps to facilitate adherence to treatment by making exercise more enjoyable, decreasing pain and fear of movement, and providing feedback and monitoring.^9,20,25,26^ Although VR therapy has demonstrated promising results in the treatment of musculoskeletal pain and in promoting a range of health benefits,^22,27–30^ it has not yet been integrated into clinical practice. This may be due to lack of awareness of the potential benefits of VR, the relatively high costs of the hard-and software, and limited evidence about the effects and treatment protocols. Before introducing VR on a large scale Birckhead et al.^31^ recommend that VR intervention development should begin with direct input from both provider and patient end-users to optimize human-centered design. In this way, acceptability of VR may be determined and potential barriers and facilitators identified. Only limited information pertaining to acceptability of VR technology is available, and as such it is timely to explore physiotherapists experiences and perspectives of immersive VR as an intervention tool for the treatment of patients with musculoskeletal shoulder pain. One qualitative study was conducted in Ireland in 2021–2022^32^ reporting that Irish physiotherapists consider VR as an appropriate tool for enhancing movement and physical activity in patients with shoulder pain. However, concerns were expressed about safety and practicalities for clinical use. It is unclear whether these results may be extrapolated to physiotherapists in other countries with differences in healthcare system, resources, accessibility, and socio-economic situation. The aim of the current study was to understand the experiences and perspectives of physiotherapists working in the Netherlands related to the use of immersive VR in the treatment of shoulder pain conditions. Both research teams investigated VR using a head mounted display (HMD) with different software. Similar results in both studies would build stronger and more generalizable evidence of perspectives and experiences of physiotherapists related to the use of VR in clinical practice.

## Methods

A qualitative, interpretive study design was conducted to explore the experiences and perspectives of physiotherapists in the Netherlands of using immersive VR in the rehabilitation of people with musculoskeletal shoulder pain.^33^ Standards for reporting qualitative research were followed (COREQ).^34^ Ethical approval was granted by the HAN University of Applied Sciences Nijmegen Ethics Committee (approval number ECO 401.10/22), Netherlands. Prior to the interview, written informed consent was obtained from each respondent. Audio recordings and transcripts of the interviews were stored separately from any information that could personally identify the participant. Audio recordings and transcripts of the interviews were stored separately from the respondents’ names and identifiers on a hard-drive which only researchers had access.

### Participants selection and recruitment

Purposive sampling was performed from September until October 2022 to recruit physiotherapists who have a minimum of 3 years’ experience working with musculoskeletal pain conditions and working with individuals with shoulder pain on a regular basis (minimum 10% total caseload). Participants were recruited via e-mail from a regional network of physiotherapists, experienced in shoulder rehabilitation (www.wseg.nl), working in primary care and in different socio-demographic environments. Physiotherapists experienced the VR device at home prior to the focus group (FG) interview. To ensure safety, the following exclusion criteria were applied for participation: a history of motion-sickness, seizures, severe vertigo or vestibular impairment. Eligible participants were sent (via email) an information sheet, outlining the study background, aims, and design, and a consent form.

### Study setting and research group

The FG interviews were conducted at Sports Medical Centre Papendal, Arnhem, The Netherlands. Interviews were conducted by two physiotherapist researchers Beate Dejaco (BD) and Anne Tankink (AT), both females. BD (FT, SFT, MT MSc) is a senior musculoskeletal physiotherapist, working part-time in clinical practice (24 years’ clinical experience) and part-time as lecturer at HAN University of Applied Sciences, master's programme in musculoskeletal rehabilitation. This is her second qualitative research project in addition to completing a two-days course in qualitative research. AT is a physiotherapist in clinical practice and master's student who assisted BD in collecting and analyzing data. Prior to commencement of the FG, basic demographic information (gender, age, number of years’ clinical experience, current clinical setting, geographical location, and previous experience of using VR, whether for clinical or entertainment purposes) was collected via email from the participants. Participants were provided with a VR head mounted display (HMD) 3™ (Pico Technology Co. Ltd, San Francisco, USA) for use at home prior to the FG interviews. They received a custom-made user manual, sent by email, designed for this study to guide participants on charging, wearing, and using the equipment. They were asked to spend approximately 20 min each day for five days familiarizing themselves with the technology and to discontinue use if they experience adverse effects e.g., motion sickness and to inform the research team. Participants were required to explore the SYNCVR fit and SYNCVR relax applications (SyncVR Medical B.V. Utrecht, The Netherlands) designed for healthcare purposes. An overview of the programs used is available: https://www.youtube.com/watch?v=9zwSzrSwqBY. They were asked to explore all available applications on the HMD, for example, virtually picking fruit, throwing objects, playing tennis, punching boxes, and collecting fireflies. They were also asked to experience applications developed for breathing exercises and relaxing techniques. Overall, the applications involved movement, concentration, and relaxation exercises.

### Data collection and processing

Three FG interviews were conducted with physiotherapists experienced in treating patients with musculoskeletal shoulder conditions. Physiotherapists were randomly allocated to the different FG groups. The interviews were scheduled to last approximately 60–90 min. With permission from participants, FG interviews were audio recorded using a Dictaphone (Zoom, H2N, The Zoom corporation, Tokyo, Japan). Nobody else was present except participants and researchers. BD used an interview-guide to monitor the interview while AT wrote field notes and ensured continuity of the recordings. On completion of the FG interview, the recorded data were stored according to FAIR principles.^35^ A detailed summary of the interview was sent to participants via email to check whether this reflected the views of participants. Participants agreed on the content and had no remarks.

### Reflexive practice

The two researchers BD and AT participated in a reflexive practice, prior to data collection and following each focus group interview. A reflection diary was used to document individual researchers’ own relationship to the research topic and the participants as well as initial thoughts regarding codes and themes. This helped to enhance quality by identifying any potential biases that may influence data collection or analysis.^36^

### Data analysis

A qualitative interpretive analysis of the data was carried out. Qualitative interpretation was chosen for this research question as it aims to report experiences, meanings and the reality of participants using their own language.^37^ It is a method of describing and interpreting data during the process of selecting codes and constructing themes.^29^ Data were analyzed within a timeframe of two weeks after each FG interview to allow early identification of codes and to determine data saturation. After the third interview data saturation was achieved as determined by the two researchers. No repeat interviews were needed. The three interviews were transcribed using Amberscript (Amberscript b.v. Amsterdam, The Netherlands), a software tool using artificial intelligence to transcribe audio records, and were then corrected manually by the researchers. Interview transcripts were then analyzed by BD and AT. A six-phase process of thematic analysis was carried out to identify “patterns or themes within datasets”^37–39^ (Table 1). Atlas Ti Qualitative Data Analysis software (© 2002–2023Atlas-ti.23 for Mac, Scientific Software Development GmbH, Berlin, Germany) was used to facilitate thematic analysis. After each phase of data analysis, BD debriefed the remaining members of the research team (co-authors) on progress and all contributed to the analysis and sorting of codes and generation of initial themes.

**Table 1.** Thematic analysis process, adapted from Braun and Clarke.^37^

Phase 1	Familiarizing with data, including correcting the auto-translated transcript, reading and re-reading the transcripts, identifying first codes
Phase 2	Generating initial codes. A code is defined as the most basic segment of the transcript meaningful to the research question. Collecting the data that is relevant to those codes.
Phase 3	Searching for themes. Organizing codes into potential themes of broader significance
Phase 4	Reviewing themes. Deciding if individual themes fit meaningfully within the data set and deciding whether themes accurately and adequately represent the entire body of data by re-reading datasets.
Phase 5	Defining and naming themes. Creating definition and narrative description of each theme.
Phase 6	Reporting analysis. Writing up final analysis and description of findings.

### Trustworthiness

To improve credibility, the following strategies were followed. First, an interview guide was created in advance with items the researchers aimed to address in all FG interviews to ensure that the research question was addressed. Second, an informal setting was created, and participants were reassured that no answers were incorrect, and that all information was important and relevant. Third, the transcripts were analyzed by two researchers (BD and AT) independently. Fourth, a detailed description of the participants and the setting was provided to enhance transferability of the outcomes. Fifth, dependability and confirmability of the outcomes are addressed by describing the study methodology and reflexivity of the researchers and by analyzing data by using the 6-phases approach as described by Braun and Clark.^37^

## Results

Participant characteristics are outlined in Table 2. A total of 20 physiotherapists were included in the study, 17 participants were divided into three FG's (three participants dropped out of the study due to logistical issues).

**Table 2.** Participants characteristics.

	Physiotherapists Mean (Standard Deviation)
	n = 17
Female	53.0%
Age in years (SD)	43.3 (11.9)
Working experience- years	18,.8 (11.5)
Experience with shoulder patients – years	5.4 (3.3)

Overall, the analysis identified three main themes: (1) VR as an extension of contemporary physiotherapy practice, (2) physiotherapist uncertainties of future care using VR (3) physiotherapist's requirements in order to incorporate VR in clinical practice, and seven subthemes. (Table 3)

**Table 3.** Three main themes and seven subthemes were identified during focus group interviews.

1. VR as an extension of contemporary physiotherapy
1.1 Positive impressions expressed by physiotherapists after experiencing immersive VR
1.2. Shoulder conditions physiotherapists would address with VR and why they think it might work
1.3. Applicability of VR in daily physiotherapy care
2. Physiotherapist uncertainties of future care using VR
2.1 The new role of the physiotherapist (less therapist-patient contact, less control on treatment process)
2.2 Unpredictability of financial burden
3. Physiotherapist's requirements in order to incorporate VR in clinical practice
3.1 The need for evidence regarding dosage, frequency, effectivity of VR
3.2 The need for practical guideline

### Theme 1. VR as an extension of contemporary physiotherapy practice

#### Positive impressions expressed by physiotherapists after experiencing immersive VR

Physiotherapists shared their experiences using immersive VR at home and the overwhelming effect of being immersed in a different world. When asked about their first impressions of using the novel technology participants shared their positive experiences when using the head mounted display as they were imagining the use of it in rehabilitation of their shoulder patients. “I found it super fun, actually found that you're immediately in that other world. say you put that thing on…. and you're immediately gone” (PT1). Participants perceived the virtual graphics as realistic and transmitting a positive feeling. They imagined patients would exercise more because they perceived VR more as playing a game rather than an exercise. “I am thinking now…the patients that don't want to practice…you don't need to practice, go play a game!” (PT6).

#### Shoulder conditions physiotherapists would address with VR and why they think it might work

Physiotherapists shared that the shoulder conditions they would treat with this novel technology would be chronic pain patients, post-operative shoulders, frozen shoulders, and patients with kinesiophobia.

“I can imagine though in people who are in really bad pain and have a lot of movement restriction”. (PT12).

Not only did they imagine the different shoulder conditions they would treat with VR, some phsyiotherapists also shared their ideas about potential working mechanisms. To them, VR seemed to trick the brain and enhance unconscious movement so that patients could move more, higher, further than they could in their real world.

“Yeah pretty cool how you can actually fool your brain”(PT6).

Participants explained that not seeing the shoulder in space alters spatial perception and therefore might decrease fear of moving the shoulder.

“That a certain position of the arm in the virtual space is not associated with pain anymore.”(PT10).

Also, more than one participant experienced altered temporal perception, they realized that they lost track of time being in the virtual world.

“I think at that moment you are not aware of time and where you are and how fast everything is moving” (PT10).

They wondered if these altered perceptions could be described as distraction but could not find consensus during the discussion. When asked, they agreed that distraction plays an important role, especially in pain management. Most physiotherapists believed that, when altered movement is captured with a camera, VR could be used as a feedback-tool to show patients that it is safe to move and that the shoulder is able to move better than expected.

“Yes, as a feedback tool and to show that, when coming back after such a long period of pain and the feeling that it's never going to work, it's never going to get better… this could be a nice tool to show that there are still things that can change” (PT15).

#### Applicability of VR in daily physiotherapy care

When discussing the applicability and feasibility of immersive VR an intense discussion arose about whether VR should be used at home or within the clinical environment.

“What I’m still struggling with a bit is will you give the head mounted display to use at home? As a homework exercise? …Or are you going to treat them, say in the clinic with the therapist beside the patient? Or do you let patients practice by themselves independently in the practice? “(PT1)

### Theme 2. Physiotherapist uncertainties of future care using VR

#### The new role of the physiotherapist

Physiotherapists discussed the role of VR in their clinic and how it could change their contemporary practice. Opinions contrasted while discussing this item; some physiotherapists imagined using VR as a stand-alone intervention to exercise at home, others felt that VR reduces therapeutic alliance and that was perceived as an unfavorable consequence.

“ If you say: go home and practice by yourself, you then reduce the therapist-patient contact…. whether it's bad? It depends, if you have a patient who is going to recover better with half an hour of your attention every week, more than from moving…. trusting the therapist is key to your rehabilitation (PT6).

Some physiotherapists shared their reluctance on letting patients exercise all by themselves and letting go of the control they still feel is imperative. They also felt that physical contact is necessary to build trust and therapeutic alliance which wouldn’t happen with a VR device at home. When discussing VR as a home exercise tool they showed that VR might create a new role for physiotherapists and that they find it exciting and at the same time frightening.

“..for you it is exciting, for me it means I do let go a world and I go, I step into another world in terms of being a therapist . I don’t have much experience with it yet, so this is quite a thing for me…” (PT8).

Physiotherapists discussed the role of VR in their clinic and how it could change their contemporary practice. Visions diverted discussing these items as some physiotherapists imagined using VR as an adjunct intervention to their usual care while others were debating that VR could be used as a stand-alone intervention. Physiotherapists shared that it would be useful to join patients within the virtual world in order to see what patients are doing in the VR world and to guide their movements

“then you can just say go to the right or go to the left…. that you can just control it”.

Others argued that exercising at home without a therapist would enhance self-management.

“ it makes much more sense to me that one practices at home every day. Then you don't let someone come to your practice every day.(PT4)

#### Unpredictability of financial burden

When discussing the new role of the physiotherapist incorporating e-health technologies such as VR in their clinical practice, uncertainties also arose regarding the financial burden when buying multiple VR HMD's and software licenses. Physiotherapists wondered how they would deal with the high costs of hard-and software purchases…

“ In the context of financing, of course, you can purchase anything, deduct it from taxes, but then of course, you also have to maintain things, then they break down. In terms of insurance, if you want to keep things running smoothly, you may need to say that if I lend things out, I need to earn some money….”(PT11).

Physiotherapists also questioned how they would manage scenarios where patients would return demolished devices or fail to return them. Some physiotherapists had learned from colleagues that lending VR devices out to patients can be tricky. Patients might not return the loaned devices or return them late or demolished which has financial consequences for the owner.

“But we just know from experience that sometimes it's difficult to get in touch with people….make that appointment to return it (VR) but they don’t bring it back and you’ve promised it to someone else again…. And, of course, you can’t have 10 of those headsets at the practice either…”(P15)

### Theme 3. Physiotherapist's requirements in order to incorporate VR in clinical practice

#### The need for evidence regarding dosage, frequency, effectivity of VR

Although physiotherapists had concerns about their professional autonomy and financial consequences when purchasing the costly hard-and software, they easily imagined using this novel technology in their clinical practice. As they were visualizing the use of immersive VR when treating patients with various shoulder conditions, they identified wishes and needs regarding the implementation. They explained that they are eager to use VR in clinical practice when treating shoulder patients. In order to prescribe VR as an intervention they would need further knowledge about the effectiveness of the intervention, dosage parameters and possible working mechanisms. “Maybe you should have some kind of training.. how does it work in the brain and for me personally, I would need a bit more evidence about the effects so that I would be more confident to explain people why I prescribe VR …”(PT10)

#### The need for a practical guideline

Physiotherapists expressed the need for an instruction manual on how to use both the hardware and software so that they can explain and guide their patients in a professional way.

“Well, an algorithm or something that guides you whether you need to use in it your clinic or at home or whatever….”(PT15)

Physiotherapists also think they would use VR even more extensively if the applications could be personalized and fitted to the patients’ needs.

“I think it actually does offer more possibilities if you personalize it (VR application). If you can grab fruit even further away, if you can hack even harder with your sword.. of course that requires a bit more of your body movement again… it can actually offer more” (PT6).

## Discussion

The aim of this study was to explore the experiences and perceptions of physiotherapists based in the Netherlands regarding the use of immersive VR in patients with shoulder pain. Key themes included VR as an extension of contemporary physiotherapy, physiotherapist uncertainties about future care using VR, and the requirements of physiotherapists to incorporate VR into clinical practice. Physiotherapists believed that immersive VR enhances current physiotherapy and has the potential to transform their role and profession. They expressed their positive experiences being immersed in a virtual world and believed VR will play an important role in future care as an add-on device, standalone intervention, feedback tool or diagnostic device.

Physiotherapists recognized VR's potential to improve function and reduce pain in shoulder patients by altering temporal and spatial perception and promoting positive emotions. Similar explanations have been given by Álvarez de la Campa Crespo et al.^40^ who found that shoulder abduction and internal rotation increased after a VR session in participants with shoulder pain. They state that by providing illusory visual feedback about the arm movement, visual information might be decoupled from proprioception and nociception. However, the value of visual feedback is somewhat debatable since studies investigating the effects on pain, when looking at a manipulated size of a painful body-part, show conflicting results^19,41^

Some physiotherapists expressed uncertainties such as the financial burden when purchasing costly hard-and software and how to incorporate it in daily care could be a barrier for implementation. To incorporate VR successfully in their practice they need more knowledge about the hard-and software, about the parameters (duration of VR session, frequency, level) that should be used and the effectiveness of a VR intervention.

These findings align with a study conducted by Brady et al.^32^ which involved focus group interviews with Irish physiotherapists exploring their beliefs and perspectives on immersive VR as a rehabilitation platform for patients with shoulder pain. Similar to the Dutch physiotherapists in the current study, Irish clinicians expressed positive sentiments regarding the use of VR in treating shoulder pain patients and had comparable thoughts on patient selection, such as targeting those with chronic shoulder pain and kinesiophobia. Another similarity between the two studies was found in the concern about patients not returning the VR hardware to the clinic. Irish physiotherapists expressed logistical challenges and stress associated with loaning the devices and patients failing to return them. One area of divergence between the studies is that, unlike their Irish colleagues, Dutch physiotherapists showed minimal concerns about patient safety when using VR, either in the clinic or at home. Second, Irish physiotherapists expressed worries about patient injuries, re-injury, or symptom flare-ups, while Dutch physiotherapists were more concerned about the space required for patients to safely practice VR at home. It remains uncertain whether these contrasting findings stem from cultural discrepancies, differences in healthcare systems, or the differences in software applications. Although the setting of the study, participants background and interview guide were comparable, participants in the current study were provided with applications specifically designed and built for healthcare purposes, while Brady et al. utilized off-the-shelf applications. The healthcare applications of the current study were more adapted to patients needs and slow-paced, this could explain why Dutch physiotherapists were not afraid of flare-ups of symptoms or injuries.

Dutch physiotherapists in this study expressed concerns about how advancing technological developments, like VR, could alter their role and potentially threaten their professional autonomy. Some highlighted that reduced face-to-face contact could have a negative impact on the therapeutic alliance and consequently the effectiveness of therapy. These findings align with previous research^42^ that compared a hybrid physiotherapy program, consisting of face-to-face sessions combined with online modules called E-exercise to usual physiotherapy care in patients with hip or knee osteoarthritis. These concordant findings demonstrate that physiotherapists are struggling with the letting go of supervising and controlling the patient in the clinic versus gaining confidence in self-managing capabilities of patients. These findings are also reflected in the qualitative analysis of the mixed method study by Mayer et al.^43^ testing a VR exposure app on patients with claustrophobia. Patients scored high on the question whether the intervention should be undergone in the clinic with a therapist and at the same time they were convinced that they could conduct the intervention alone at home.

Physiotherapists highlighted the importance of acquiring comprehensive knowledge regarding the effectiveness and underlying working mechanisms of VR applications, as well as guidelines to clinical decision-making and implementation of VR in routine physiotherapy care. These comments align with the findings of De Veer et al.^44^ who conducted a qualitative study among nursing staff members from various health care sectors. Their research aimed to evaluate the introduction and implementation of new technologies in nursing care, specifically identifying barriers and facilitators to successful implementation. One of the barriers identified was the perception of technologies being ineffective or difficult to use. Addressing barriers related to VR effectiveness, implementation and adoption by physiotherapists needs close collaboration among stakeholders such as hardware suppliers, software developers, healthcare economists, research departments, clinicians, patient groups, and funders.

### Strengths and limitations

An important strength of this study is the similarity to the study design conducted by Brady et al.^32^ Results may therefore be compared and stronger conclusions may be drawn regarding the experiences and perceptions of physiotherapists on the use of VR in physiotherapy care. Another strength is that participants were experienced in the rehabilitation of individuals with shoulder pain. As a result, they were highly capable of visualizing the possibilities, needs and concerns regarding the use of VR when treating patients with diverse shoulder pathologies. A limitation was that participants had only experienced VR for themselves rather than using VR as an intervention tool in patients with shoulder pain. Experiences are therefore limited and might differ once physiotherapists use VR in clinical practice and gain further insight including patient feedback and experience regarding the practicalities of the VR device. Another limitation is that focus group interviews have a social desirability bias. However, the researchers strived for an equal response of participants by asking direct questions to the participants who were more quiet and to dig deeper when needed.

### Future research

Future research should focus on providing evidence about the effects and optimal dosage to assist clinicians with treatment parameters when treating patients with shoulder pain. Before investigating VR in large scale randomized trials, case series and pilot studies should explore early clinical efficacy and acceptability. To ensure financial viability, business models should be calculated and practical guidelines to assist clinicians in the clinical decision-making process.

## Conclusion

This study explored physiotherapist's experiences of VR for treating people with shoulder pain. Physiotherapists were interested in the mechanisms by which the technology might support practice. They expressed positive and negative opinions regarding VR. One potential benefit was supporting exercise therapy. Concerns about how their professional role and identity might change with VR were a negative concern. Clinicians described their own requirements to support the introduction of VR into clinical practice. These findings provide insight into clinician acceptability of VR as an assessment and intervention tool. They inform stakeholders such as researchers and content developers on the design of future research protocols and healthcare applications particularly for managing musculoskeletal shoulder pain.

## Supplemental Material

sj-docx-1-dhj-10.1177_20552076241234738 - Supplemental material for Experiences of physiotherapists considering virtual reality for shoulder rehabilitation: A focus group study Supplemental material, sj-docx-1-dhj-10.1177_20552076241234738 for Experiences of physiotherapists considering virtual reality for shoulder rehabilitation: A focus group study by Beate Dejaco, Niamh Brady, Anne Tankink, Jeremy Lewis, Harry van Goor, J Bart Staal and Niki Stolwijk in DIGITAL HEALTH

sj-docx-2-dhj-10.1177_20552076241234738 - Supplemental material for Experiences of physiotherapists considering virtual reality for shoulder rehabilitation: A focus group study Supplemental material, sj-docx-2-dhj-10.1177_20552076241234738 for Experiences of physiotherapists considering virtual reality for shoulder rehabilitation: A focus group study by Beate Dejaco, Niamh Brady, Anne Tankink, Jeremy Lewis, Harry van Goor, J Bart Staal and Niki Stolwijk in DIGITAL HEALTH

sj-jp2-3-dhj-10.1177_20552076241234738 - Supplemental material for Experiences of physiotherapists considering virtual reality for shoulder rehabilitation: A focus group study Supplemental material, sj-jp2-3-dhj-10.1177_20552076241234738 for Experiences of physiotherapists considering virtual reality for shoulder rehabilitation: A focus group study by Beate Dejaco, Niamh Brady, Anne Tankink, Jeremy Lewis, Harry van Goor, J Bart Staal and Niki Stolwijk in DIGITAL HEALTH

sj-jp2-4-dhj-10.1177_20552076241234738 - Supplemental material for Experiences of physiotherapists considering virtual reality for shoulder rehabilitation: A focus group study Supplemental material, sj-jp2-4-dhj-10.1177_20552076241234738 for Experiences of physiotherapists considering virtual reality for shoulder rehabilitation: A focus group study by Beate Dejaco, Niamh Brady, Anne Tankink, Jeremy Lewis, Harry van Goor, J Bart Staal and Niki Stolwijk in DIGITAL HEALTH

## References

[1] Picavet HSJ Schouten JSAG . *Musculoskeletal pain in the Netherlands: Prevalences, consequences and risk groups, the DMC 3-Study* . 10.1016/s0304-3959(02)00372-x 12620608

[2] Dean E Söderlund A . What is the role of lifestyle behaviour change associated with non-communicable disease risk in managing musculoskeletal health conditions with special reference to chronic pain? BMC Musculoskelet Disord 2015; 16. doi:10.1186/s12891-015-0545-y PMC4397667 25888381

[3] Ulack C Suarez J Brown L , et al. What are people that seek care for rotator cuff tendinopathy experiencing in their daily life? J Patient Exp 2022; 9: 237437352110698. 10.1177/23743735211069811 PMC8744205 35024444

[4] Dejaco B Habets B van Loon C , et al. Eccentric versus conventional exercise therapy in patients with rotator cuff tendinopathy: a randomized, single blinded, clinical trial. Knee Surg Sports Traumatol Arthrosc 2017; 25: 2051–2059. 27351548 10.1007/s00167-016-4223-x

[5] Holmgren T Hallgren HB Öberg B , et al. Effect of specific exercise strategy on need for surgery in patients with subacromial impingement syndrome: randomised controlled study. BMJ (Online) 2012; 344: e787. 10.1136/bmj.e787 PMC3282676 22349588

[6] Maenhout AG Mahieu NN de Muynck M , et al. Does adding heavy load eccentric training to rehabilitation of patients with unilateral subacromial impingement result in better outcome? A randomized, clinical trial. Knee Surg Sports Traumatol Arthrosc 2013; 21: 1158–1167. 22581193 10.1007/s00167-012-2012-8

[7] Schydlowsky P Szkudlarek M Madsen OR . Comprehensive supervised heavy training program versus home training regimen in patients with subacromial impingement syndrome: a randomized trial. BMC Musculoskelet Disord 2022; 23: 52. 35033043 10.1186/s12891-021-04969-0 PMC8760780

[8] Bailey DL Holden MA Foster NE , et al. Defining adherence to therapeutic exercise for musculoskeletal pain: a systematic review. Br J Sports Med 2020; 54: 326–331. 29875278 10.1136/bjsports-2017-098742

[9] Wiley J Meade LB Bearne LM , et al. Behaviour change techniques associated with adherence to prescribed exercise in patients with persistent musculoskeletal pain: systematic review. Br J Health Psychol 2019; 24: 10–30. 29911311 10.1111/bjhp.12324 PMC6585717

[10] Byra J Czernicki K . The effectiveness of virtual reality rehabilitation in patients with knee and hip osteoarthritis. J Clin Med 2020; 9: 2639. 32823832 10.3390/jcm9082639 PMC7465023

[11] Gokeler A Bisschop M Myer GD , et al. Immersive virtual reality improves movement patterns in patients after ACL reconstruction: implications for enhanced criteria-based return-to-sport rehabilitation. Knee Surg Sports Traumatol Arthrosc 2016; 24: 2280–2286. 25311052 10.1007/s00167-014-3374-x

[12] Gumaa M Youjssef AR . Is virtual reality effective in orthopedic rehabilitation? A systematic review and meta-analysis. Phys Ther 2019; 99: 1304–1325. 31343702 10.1093/ptj/pzz093

[13] Pekyavas NO Ergun N . Comparison of virtual reality exergaming and home exercise programs in patients with subacromial impingement syndrome and scapular dyskinesis: short term effect. Acta Orthop Traumatol Turc 2017; 51: 238–242. 28446376 10.1016/j.aott.2017.03.008 PMC6197467

[14] Piqueras M Marco E Coll M , et al. Effectiveness of an interactive virtual telerehabilitation system in patients after total knee arthoplasty: a randomized controlled trial. J Rehabil Med 2013; 45: 392–396. 23474735 10.2340/16501977-1119

[15] Stamm O Dahms R Müller-Werdan U . Virtual reality in pain therapy: A requirements analysis for older adults with chronic back pain. J Neuroeng Rehabil 2020; 17. doi:10.1186/s12984-020-00753-8 PMC7523056 32993678

[16] Thomas JS France CR Applegate ME , et al. Feasibility and safety of a virtual reality dodgeball intervention for chronic low back pain: a randomized clinical trial. Journal of Pain 2016; 17: 1302–1317. 27616607 10.1016/j.jpain.2016.08.011 PMC5125833

[17] Eijlers R Utens EMWJ Staals LM , et al. Systematic review and meta-analysis of virtual reality in pediatrics: effects on pain and anxiety. Anesth Analg 2019; 129: 1344–1353. 31136330 10.1213/ANE.0000000000004165 PMC6791566

[18] Hoffman HG Chambers GT Meyer WJ , et al. Virtual reality as an adjunctive non-pharmacologic analgesic for acute burn pain during medical procedures. Ann Behav Med 2011; 41: 183–191. 21264690 10.1007/s12160-010-9248-7 PMC4465767

[19] Matamala-Gomez M Donegan T Bottiroli S , et al. Immersive virtual reality and virtual embodiment for pain relief. Front Hum Neurosci 2019: 13. doi:10.3389/fnhum.2019.00279 PMC6736618 31551731

[20] Hoffman HG Sharar SR Coda B , et al. Manipulating presence influences the magnitude of virtual reality analgesia. Pain 2004; 111: 162–168. 15327820 10.1016/j.pain.2004.06.013

[21] Matamala-Gomez M Diaz Gonzalez AM Slater M , et al. Decreasing pain ratings in chronic arm pain through changing a virtual body: different strategies for different pain types. Journal of Pain 2019; 20: 685–697. 30562584 10.1016/j.jpain.2018.12.001

[22] Fowler CA Ballistrea LM Mazzone KE , et al. Virtual reality as a therapy adjunct for fear of movement in veterans with chronic pain: single-arm feasibility study. JMIR Form Res 2019; 3: e11266. 10.2196/11266 PMC6914277 31670696

[23] Gromala D Tong X Choo A , et al. The virtual meditative walk: virtual reality therapy for chronic pain management. In: 33rd Annual ACM conference on human factors in computing systems; 2015; 2015; ACM.

[24] Dubé MO Desmeules F Lewis JS , et al. Does the addition of motor control or strengthening exercises to education result in better outcomes for rotator cuff-related shoulder pain? A multiarm randomised controlled trial. Br J Sports Med 2023; 57: 457–463. 36796859 10.1136/bjsports-2021-105027

[25] Peek K Sanson-Fisher R Mackenzie L , et al. Interventions to aid patient adherence to physiotherapist prescribed self-management strategies: a systematic review. Physiotherapy (United Kingdom) 2016; 102: 127–135. 10.1016/j.physio.2015.10.003 26821954

[26] Triberti S Repetto C Riva G . Psychological factors influencing the effectiveness of virtual reality-based analgesia: a systematic review. Cyberpsychol Behav Soc Netw 2014; 17: 335–345. 24892195 10.1089/cyber.2014.0054

[27] Carl E Stein AT Levihn-Coon A , et al. Virtual reality exposure therapy for anxiety and related disorders: a meta-analysis of randomized controlled trials. J Anxiety Disord 2019; 61: 27–36. 30287083 10.1016/j.janxdis.2018.08.003

[28] Lier EJ De Vries M Steggink EM , et al. Systematic review and meta-analysis effect modifiers of virtual reality in pain management: a systematic review and meta-regression analysis. Pain 2023; 164: 1658–1665. 36943251 10.1097/j.pain.0000000000002883 PMC10348651

[29] Pourmand A Davis S Marchak A , et al. Virtual reality as a clinical tool for pain management. Curr Pain Headache Rep 2018; 22. doi:10.1007/s11916-018-0708-2 29904806

[30] de Vries FS van Dongen RTM Bertens D . Pain education and pain management skills in virtual reality in the treatment of chronic low back pain: a multiple baseline single-case experimental design. Behav Res Ther 2023; 162: 104257. 36731183 10.1016/j.brat.2023.104257

[31] Birckhead B Khalil C Liu X , et al. Recommendations for methodology of virtual reality clinical trials in health care by an international working group: iterative study. JMIR Ment Health 2019; 6: e11973. 10.2196/11973 PMC6374734 30702436

[32] Brady N Dejaco B Lewis J , et al. Physiotherapist beliefs and perspectives on virtual reality supported rehabilitation for the management of musculoskeletal shoulder pain: a focus group study. PLoS One 2023; 18: e0284445. 10.1371/journal.pone.0284445 PMC10104293 37058507

[33] Thompson Burdine J Thorne S Sandhu G . Interpretive description: a flexible qualitative methodology for medical education research. Med Educ 2021; 55: 336–343. 32967042 10.1111/medu.14380

[34] Tong A Sainsbury P Craig J . Consolidated criteria for reporting qualitative research (COREQ): a 32-item checklist for interviews and focus groups. Int J Qual Health Care 2007; 19: 349–357. 17872937 10.1093/intqhc/mzm042

[35] Wilkinson MD Dumontier M Aalbersberg IJJ , et al. Comment: The FAIR Guiding Principles for scientific data management and stewardship. Sci Data 2016; 3. doi:10.1038/sdata.2016.18 PMC4792175 26978244

[36] Jootun D McGhee G Marland GR . Reflexivity: promoting rigour in qualitative research. Nurs Stand 2009; 23: 42–46. 10.7748/ns2009.02.23.23.42.c6800 19263905

[37] Braun V Clarke V . Using thematic analysis in psychology. Qual Res Psychol 2006; 3: 77–101.

[38] Braun V Clarke V . What can “‘thematic analysis’” offer health and wellbeing researchers? International Journal of Qualitative Studies on Health and Well-Being; 2014; 9: 26152. 25326092 10.3402/qhw.v9.26152 PMC4201665

[39] Kiger ME Varpio L . Medical teacher thematic analysis of qualitative data: AMEE guide No. 131 thematic analysis of qualitative data: AMEE guide No. 131. Med Teach 2020; 42: 846–854. 32356468 10.1080/0142159X.2020.1755030

[40] Álvarez de la Campa Crespo M Donegan T Amestoy-Alonso B , et al. Virtual embodiment for improving range of motion in patients with movement-related shoulder pain: an experimental study. J Orthop Surg Res 2023; 18. doi:10.1186/s13018-023-04158-w PMC10523655 37752613

[41] Mancini F Longo MR Kammers MPM , et al. Visual distortion of body size modulates pain perception. Psychol Sci 2011; 22: 325–330. 21303990 10.1177/0956797611398496

[42] Kloek CJJ Bossen D de Vries HJ , et al. Physiotherapists’ experiences with a blended osteoarthritis intervention: a mixed methods study. Physiother Theory Pract 2020; 36: 572–579. 29952687 10.1080/09593985.2018.1489926

[43] Mayer G Gronewold N Polte K , et al. Experiences of patients and therapists testing a virtual reality exposure app for symptoms of claustrophobia: mixed methods study. JMIR Ment Health 2022; 9: e40056. 10.2196/40056 PMC9764154 36469413

[44] De Veer AJE Fleuren MAH Bekkema N , et al. Successful implementation of new technologies in nursing care: A questionnaire survey of nurse-users. BMC Med Inform Decis Mak 2011; 11. doi:10.1186/1472-6947-11-67 PMC3214145 22032728

