# Supplementary material for: Experiences of physiotherapists considering virtual reality for shoulder rehabilitation: A focus group study
Source: Digit Health. 2024 Feb 26;10:20552076241234738. doi: 10.1177/20552076241234738 (PMC10898295; doi:10.1177/20552076241234738)
Supplement: sj-docx-1-dhj-10.1177_20552076241234738 - Supplemental material for Experiences of physiotherapists considering virtual reality for shoulder rehabilitation: A focus group study [file sj-docx-1-dhj-10.1177_20552076241234738.docx]

Code tree
