# Supplementary material for: Experiences of physiotherapists considering virtual reality for shoulder rehabilitation: A focus group study
Source: Digit Health. 2024 Feb 26;10:20552076241234738. doi: 10.1177/20552076241234738 (PMC10898295; doi:10.1177/20552076241234738)
Supplement: sj-docx-2-dhj-10.1177_20552076241234738 - Supplemental material for Experiences of physiotherapists considering virtual reality for shoulder rehabilitation: A focus group study [file sj-docx-2-dhj-10.1177_20552076241234738.docx]

| **INTERVIEWGUIDE** | |
| --- | --- |
| Introduction will be held before the focus group interview starts | |
| **Introduction** | First of all, welcome to this focus group. How nice of you to take the time to participate in this study. The information letter I sent you contains information about the study. Do you have any questions about it beforehand? |
| **Aim of the study** | You have had several days to test the VR device. Now I would like to ask you to share your experiences with the device during this meeting. We are also interested in your perceptions. |
| **Informed consent** | To conduct the interview, I need your permission to process and analyse the audio recordings, will you give me your permission to do so? |
| Topics shared below will not be shared with participants in advance. This is to avoid unnecessarily externally directing the natural course of the conversation. If topics are not discussed in this way, specific questions will still be asked. | |
| **Topics** | 1. Entertainment 2. manageability 3. applicability 4. cohort to treat 5. safety     When other topics arise due to the natural course of the conversation, we will add them. There is plenty of time to discuss and gather ideas freely. |
|  | |
|  | |
| **Topic 1. Entertainment** | |
| - motivation - fun | - How did you feel about using VR? |
| **Topic 2. manageability** |  |
| - Set up - The use - charge | - How was the usability of the VR device? - How did you experience storage and charging? - In relation to patients: How do you think they will experience it? - As a therapist, what barriers do you see in the practice situation regarding the device's usability for patients? |
| **Topic 3. applicability** |  |
| - Side effects - VR deployment in practice - Aspects of VR - Facilitators - Barriers | - Do you see a role for VR in the research and rehabilitation of patients with shoulder pain? - If so, which aspect of VR has the potential to be most effective? (Training/movement, distraction, engagement, motivation, competition, reward, education, exposure?) - How do you feel about using VR for: Training/movement, distraction, engagement, motivation, competition, reward, education, exposure? - What would make it easy to start using VR in practice? - What hurdles do you anticipate when implementing VR in practice? - What would hold you back? And how might these be avoided? - Have you experienced any side effects during or after using VR? |
| **Topic 4. Cohort to treat** |  |
| - Subgroups - Shoulder patients | Welke specifieke subgroep binnen de patiënten met schouderpijn denken jullie zal goed reageren op een VR interventie? |
| **Topic 5. safety** |  |
|  |  |
|  | - What are your experiences regarding safety? - How will the patient experience this? |
|  |  |
|  |  |

Adaptations after 2^nd^ interview:

Topics added: space, the role of insurance companies.
